# Decision Tree Analysis of Traditional Risk Factors of Carotid Atherosclerosis and a Cutpoint-Based Prevention Strategy

**DOI:** 10.1371/journal.pone.0111769

**Published:** 2014-11-14

**Authors:** Guangming Qin, Laisheng Luo, Lihong Lv, Yufei Xiao, Jiangfeng Tu, Lisha Tao, Jiaqi Wu, Xiaoxiao Tang, Wensheng Pan

**Affiliations:** 1 Department of Laboratory, The Second Affiliated Hospital, School of Medicine, Zhejiang University, Hangzhou, China; 2 Department of Gastroenterology, The Second Affiliated Hospital, School of Medicine, Zhejiang University, Hangzhou, China; 3 Department of Gastroenterology, The Second Affiliated Hospital Binjiang Campus, School of Medicine, Zhejiang University, Hangzhou, China; University of Milan, Italy

## Abstract

**Background:**

Reducing the exposure to risk factors for the prevention of cardio-cerebral vascular disease is a crucial issue. Few reports have described practical interventions for preventing cardiovascular disease in different genders and age groups, particularly detailed and specific cutpoint-based prevention strategies.

**Methods:**

We collected the health examination data of 5822 subjects between 20 and 80 years of age. The administration of medical questionnaires and physical examinations and the measurement of blood pressure, fasting plasma glucose (FPG) and blood lipids [total cholesterol (TC), triglycerides (TG), high density lipoprotein–cholesterol (HDL-C), and low density lipoprotein-cholesterol (LDL-C)] were performed by physicians. Carotid ultrasound was performed to examine the carotid intima-media thickness (CIMT), which was defined as carotid atherosclerosis when CIMT ≥0.9 mm. Decision tree analysis was used to screen for the most important risk factors for carotid atherosclerosis and to identify the relevant cutpoints.

**Results:**

In the study population, the incidence of carotid atherosclerosis was 12.20% (men: 14.10%, women: 9.20%). The statistical analysis showed significant differences in carotid atherosclerosis incidence between different genders (*P*<0.0001) and age groups (*P*<0.001). The decision tree analysis showed that in men, the most important traditional risk factors for carotid atherosclerosis were TC (cutpoint [CP]: 6.31 mmol/L) between the ages of 20–40 and FPG (CP: 5.79 mmol/L) between the ages of 41–59. By comparison, LDL-C (CP: 4.27 mmol/L) became the major risk factor when FPG ≤5.79 mmol/L. FPG (CP: 5.52 mmol/L) and TG (CP: 1.51 mmol/L) were the most important traditional risk factors for women between 20–40 and 41–59 years of age, respectively.

**Conclusion:**

Traditional risk factors and relevant cutpoints were not identical in different genders and age groups. A specific gender and age group-based cutpoint strategy might contribute to preventing cardiovascular disease.

## Introduction

Numerous epidemiological studies have shown that the incidence of cardiovascular and cerebrovascular diseases is increasing. Preventive interventions should be conducted for patients with subclinical disease because reducing the exposure to risk factors has been shown to be most effective during this stage [1], [2]. There are few reports describing the epidemiological features of traditional risk factors in different genders and age groups as well as the relevant cutpoints for risk factors. In most studies, logistic regression analysis has been used to screen for the important risk factors after adjustment for gender and age. Gender and age are unmodifiable risk factors for cardio-cerebral vascular disease, and confounding could occur through collinearity in a logistic regression in cases in which multiple risk factors are involved. Decision tree analysis could overcome such disadvantages [3], [4], screen for the most important risk factors for cardiovascular diseases in different genders and age groups and identify the cutpoints for risk factors. (A decision tree is a flowchart-like figure in which an internal node represents a “test” of an attribute, each branch represents the outcome of the test and each leaf node represents a class label; the paths from the root to the leaf represent classification rules. Decision trees are regularly used in operations research, specifically in decision analysis, to help identify a strategy most likely to reach a goal). The etiology of cardiovascular disease has been shown to be atherosclerosis [5]–[8]. Carotid intima-media thickness (CIMT) is a sensitive indicator of early stage atherosclerosis [7]–[10], and CIMT testing has good consistency with assessments using medical ultrasonic equipment and histopathological examinations [8], [9], [11]. CIMT testing could be used for noninvasive assessment or for monitoring systemic atherosclerosis and to warn of cardiovascular and cerebrovascular events [9], [11], [12]. Many risk factors play a role in atherosclerosis. Studies [6], [13]–[16] have suggested that traditional risk factors including blood pressure, hyperglycemia, dyslipidemia, age and gender play an important role in more than 50% of atherosclerosis development. This study aimed to examine the incidence of carotid atherosclerosis in healthy people, to screen for the most important risk factors in different genders and age groups and to suggest a cutpoint-based prevention strategy using decision tree analysis.

## Methods

### 1. Ethics Statement

This study was reviewed and approved by the institutional review board (IRB) of the Second Affiliated Hospital of the Zhejiang University School of Medicine (ethical review code: Research 2014–113). The need to obtain written informed consent from the subjects (or from the next of kin/caregiver in the case of children) for use of the clinical records of the participants was waived by the IRB for this retrospective study. With the approval of the IRB, we used the patient identification numbers to collect and analyze the clinical records. The names and other personal information were anonymized and de-identified prior to analysis to protect patient privacy.

### 2. Subjects

We collected the data of 5822 subjects, between the ages of 20 and 80, who came for a health examination at the Second Affiliated Hospital of the Zhejiang University School of Medicine from September 2011 to December 2012. Most of the subjects were from Zhejiang province. In total, 4910 subjects were included in the statistical analysis after the exclusion of the subjects with a history of cardiovascular and cerebrovascular diseases, cancer, diabetes, severe mental disorders, chronic kidney disease [17], acute and chronic liver disease, infectious diseases, and acute and chronic fever of unknown origin as well as the subjects undergoing drug therapy for hypertension, diabetes mellitus, coronary heart disease and hyperthyroidism and those who consumed excessive alcohol [18]. The patients were divided into 20–40, 41–59 and 60–80 age groups. Within these groups, there were 3047 men (45.13±9.06 years) and 1863 women (44.91±9.60 years).

### 3. Questionnaires and physical examination

Physicians administered the medical questionnaires, conducted the physical examinations and measured the blood pressure of the patients. For the study, we measured height and weight to calculate the body mass index [BMI  =  weight (kg)/height ^2^ (m)] of the patients. Blood pressure was measured using a standardized method in which the systolic and diastolic blood pressures of the right arm were measured using an automated device (Omron 711, USA) after having the subjects rest for 5 minutes in a sitting position. The mean of the two consecutive blood pressure measurements was recorded.

### 4. Laboratory tests

The subjects fasted for 12 hours and had venous blood samples taken to measure their fasting plasma glucose (FPG) level and blood lipid levels (total cholesterol: TC, triglycerides: TG, high density lipoprotein–cholesterol: HDL-C, low density lipoprotein-cholesterol: LDL-C). FPG was measured using the hexokinase (HK) method, and TC was measured by the cholesterol oxidase-peroxidase method (CHO-POD). TG was measured by the glycerol phosphate oxidase-peroxidase method. HDL-C was measured by the direct-surfactant clearance method, and LDL-C was measured by the direct method-selected inhibitor method with a Beckman Coulter Chemistry Analyzer AU5400 (Beckman Coulter, Mishima K.K, Tokyo, Japan). To guarantee the accuracy and comparability of the results, two quality control materials (plasma glucose: lot number: 45641 and 45643; lipids: 57251 and 57252, products of BIO-RAD) of different concentrations were tested every day. The cumulative coefficients of variation were as follows: FPG (1.83%; 1.73%), TG (4.0%; 2.56%), TC (2.05%; 1.92%), HDL-C (2.60%; 2.70%) and LDL-C (3.03%; 3.01%).

### 5. Carotid ultrasound measurement

The distal segment and stigma compartments of the cephalic artery and the proximal segment of the internal carotid artery were measured on both sides [19]. Each blood vessel was measured in three sections within a range of 1 cm in the proximal wall and distant from the sidewalls. The subject was determined to have carotid atherosclerosis when the CIMT ≥0.9 mm [20], [21]. The physicians scanned and stored the images using standardized operating procedures.

### 6. Statistical analysis

All of the data were analyzed using the IBM SPSS 20.0 software package. The continuous data were compared using *t*-tests, whereas the discrete count data were compared with the χ2 test. Decision tree analysis was used to assess the incidence of carotid atherosclerosis and to screen for the most important relevant traditional risk factors and cutpoints. Statistical significance was determined at *P*<0.05.

## Results

### 1. The distribution characteristics of carotid atherosclerosis in the studied population

**Figure 1:**
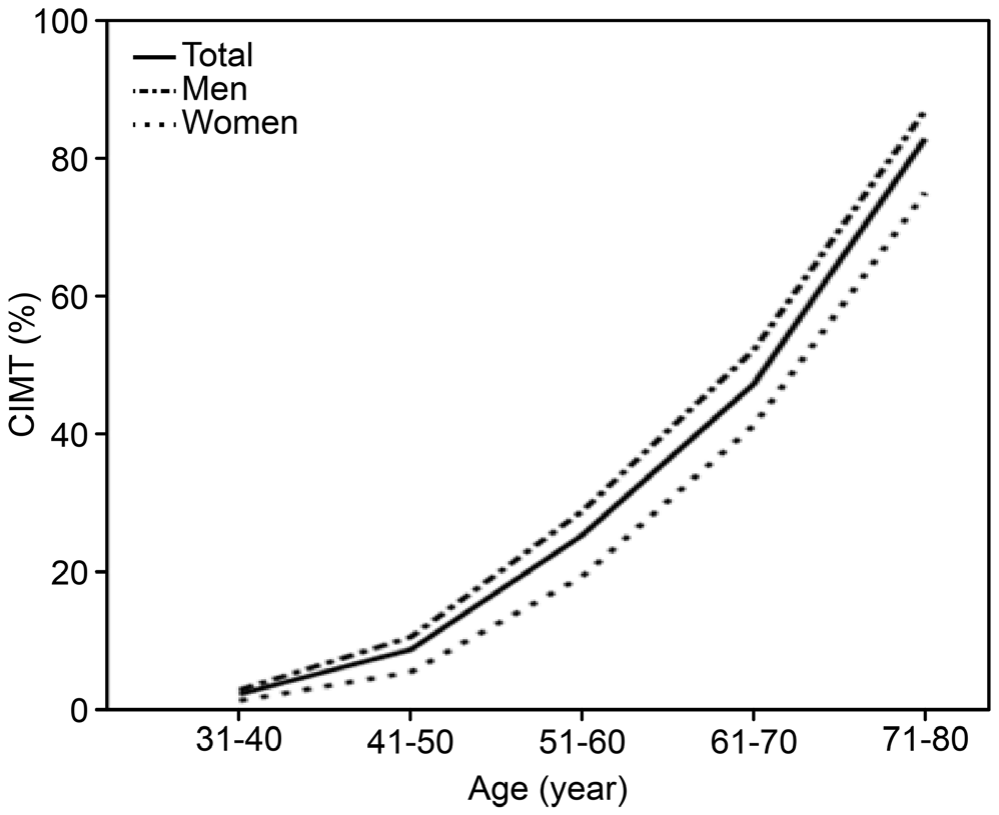
The incidence of carotid atherosclerosis in different age groups and genders.

The incidence of carotid atherosclerosis in the studied population was 12.20% (men: 14.10%, women: 9.20%). Statistically, the incidence was higher in men than in women (χ^2^ = 25.89, *P*<0.0001). The incidence according to gender and age group is presented in Figure 1. The results show that the incidence increases with age. This mathematical relationship is expressed by the following quadratic curve equations: CIMT% = 7.23–10.66*age + 3.98*age^2^ in men, and CIMT% = 11.79–14.68*age + 4.17*age^2^ in women.

### 2. The comparison of traditional risk factors between genders

The comparison of the traditional risk factors (blood pressure, blood lipids, blood glucose and age) between the genders is presented in Table 1. The differences between the genders in blood pressure, blood lipids and blood glucose were statistically significant (*P*<0.001).

**Table 1:** The comparison of traditional risk factors between genders (mean ± SD).

Traditional risk factors	M (3047) Mean SD		W (1863) Mean SD		*t*	*P*
Age (years)	45.13	9.06	44.91	9.60	0.804*	0.421
SBP (mmHg)	129.77	13.83	120.68	13.87	22.308*	<0.001
DBP (mmHg)	80.83	11.47	73.10	10.74	23.846*	<0.001
BMI (kg/m^2^)	24.88	3.01	22.68	2.77	26.05	<0.001
FPG (mmol/L)	5.44	1.05	5.19	0.74	9.652*	<0.001
TC (mmol/L)	5.09	0.96	4.94	0.94	5.437	<0.001
TG (mmol/L)	1.99	1.30	1.37	0.69	21.892*	<0.001
HDL-C (mmol/L)	1.37	0.31	1.60	0.34	−24.126*	<0.001
LDL-C (mmol/L)	3.27	0.79	2.93	0.76	14.96	<0.001

*: Using *t*'-test when equal variances were not assumed.

Abbreviations: M: men; W: women; SBP: systolic blood pressure; DBP: diastolic blood pressure; BMI: body mass index; FPG: fasting plasma glucose; TC: total cholesterol; TG: triglyceride; HDL-C: high-density lipoprotein-cholesterol; LDL-C: low-density lipoprotein-cholesterol; SD: standard deviation.

### 3. Decision tree analysis on traditional risk factors of carotid atherosclerosis

**Figure 2:**
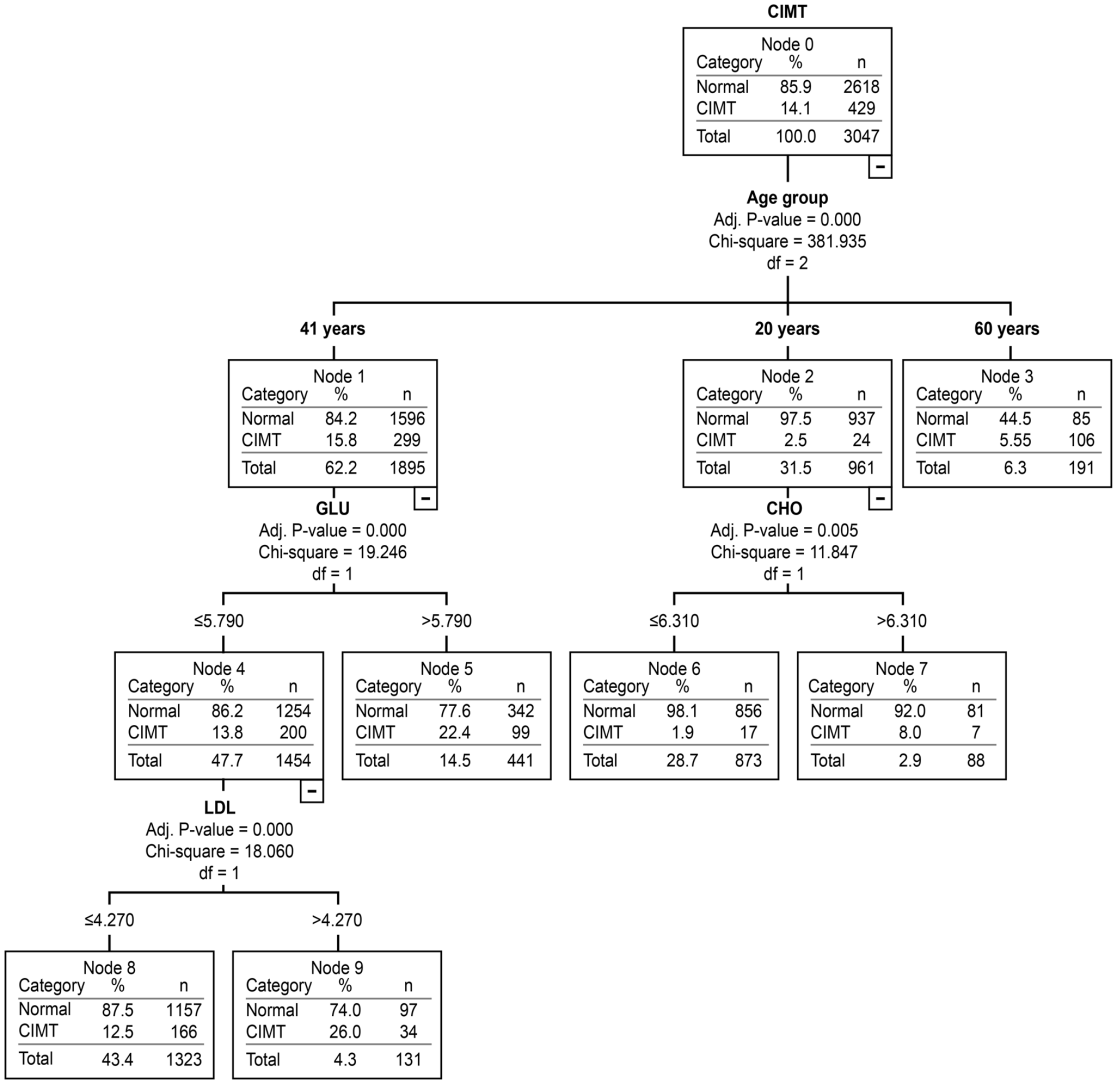
The assessment of traditional risk factors in men for carotid atherosclerosis in different age groups using decision tree analysis.

**Figure 3:**
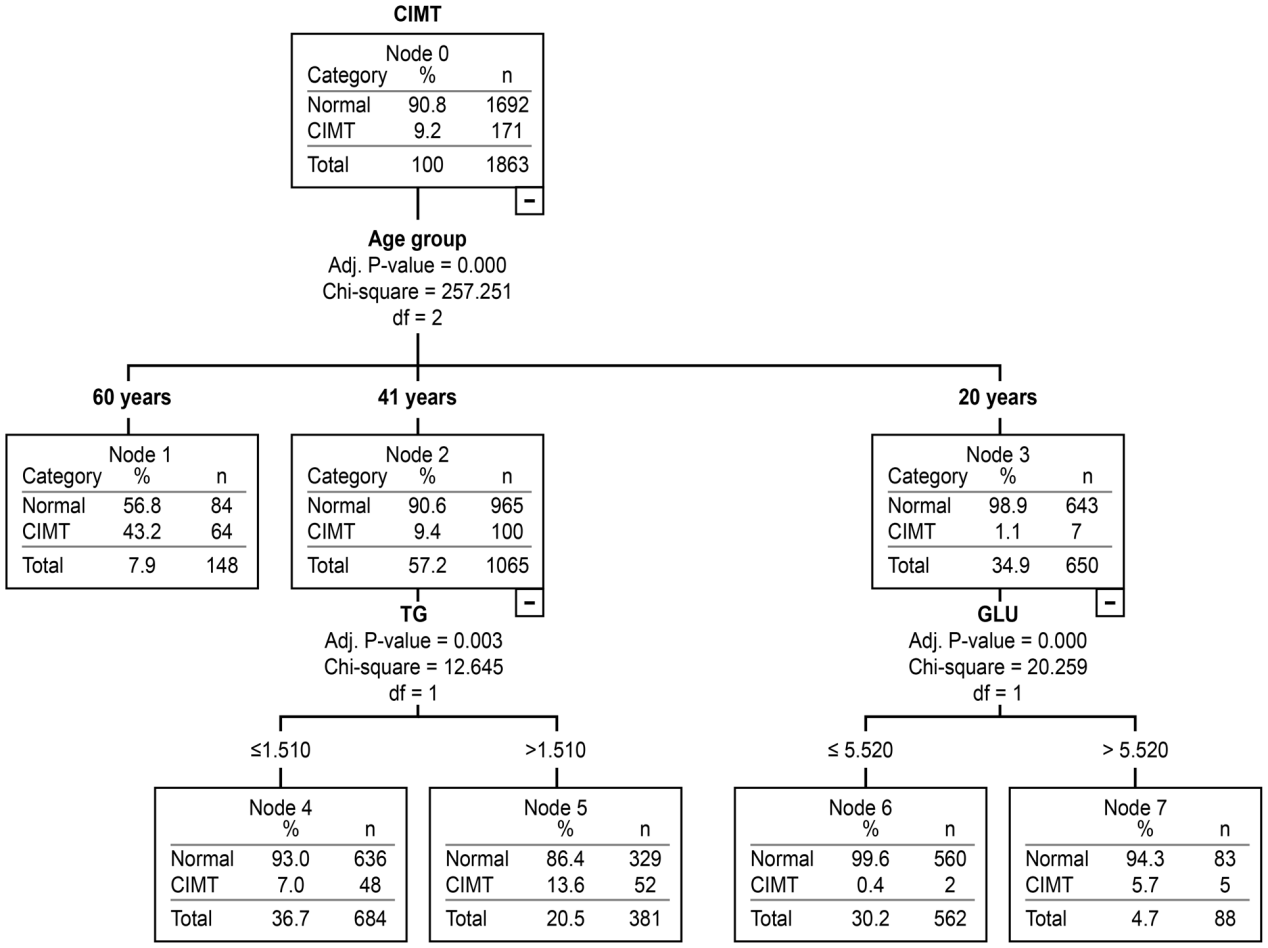
The assessment of traditional risk factors for carotid atherosclerosis in women in different age groups using decision tree analysis.

We used decision tree analysis to screen for the most important traditional risk factors for carotid atherosclerosis by gender and age groups. The results are presented in Figure 2 and Figure 3.

As observed in Figure 2, the most important traditional risk factor for carotid atherosclerosis in men was TC (CP: 6.31 mmol/L) in the 20–40 year age group and FPG (CP: 5.79 mmol/L) in the 41–59 year age group. LDL-C level (CP: 4.27 mmol/L) became the most important risk factor when the FPG ≤5.79 mmol/L. The most important traditional risk factor for carotid atherosclerosis in women was FPG (CP: 5.52 mmol/L) between 20 and 40 years of age and TG (CP: 1.51 mmol/L) between 41 and 59 years of age (Figure 3). Between the ages of 60 and 80, the traditional risk factors showed no statistical significance regardless of gender.

## Discussion

The study showed that carotid atherosclerosis is prevalent in healthy populations (men: 14.10%, women: 9.20%, *P*<0.001). This result is consistent with those of other reports. The incidence of carotid atherosclerosis increases with age. In this study, the incidence in subjects over 60 years old was 55.5% in men and 43.2% in women, which is consistent with a study conducted by Johnsen SH [19], which reported that the incidence was 52.9% in men and 43.7% in women. The different incidences in traditional risk factors observed in this study between genders, such as the incidences of blood pressure, blood lipids and blood glucose, were shown to be statistically significant (*P*<0.001) (Table 1). Therefore, it is necessary to separate the complicated relationships between traditional risk factors and gender and age in carotid atherosclerosis.

Traditional statistical methods such as logistic regression analysis have been effectively applied and widely recognized in the screening of disease risk factors. However, logistic regression has limitations in managing complicated data because many factors are involved that could affect each other [22], [23]. Decision tree analysis might prevent such disadvantages. In this study, decision tree analysis was successfully used to screen for the most important risk factors for carotid atherosclerosis and to identify the cutpoints for risk factors by gender and age group. The results presented in Figure 2 showed that in men, the most important traditional risk factor for carotid atherosclerosis was TC (CP: 6.31 mmol/L) from 20–40 years of age and FPG (CP: 5.79 mmol/L) from 41–59 years of age. By comparison, LDL-C (CP: 4.27 mmol/L) became the major risk factor when FPG ≤5.79 mmol/L. Figure 3 demonstrates that the most important traditional risk factor for carotid atherosclerosis in women was FPG (CP: 5.52 mmol/L) from 20–40 years of age and TG (CP: 1.51 mmol/L) from 41–59 years of age. From 60–80 years of age, the traditional risk factors showed no statistical significance regardless of gender. We assume that non-traditional risk factors might have played a more important role in the subjects older than 60 years of age. The results suggest that traditional risk factors and relevant cutpoints are not identical in different genders and age groups [24]. This specific gender and age group-based cutpoint strategy might be more effective in the prevention of cardiovascular disease.

Important conclusions were described in the 2012 China city residents' health white paper published by the Chinese Medical Doctor Association, the Chinese Hospital Association and the Beijing Health Maintenance Organization. People aged 35–65 years have become the major demographic group affected by chronic diseases, including chronic cardiovascular diseases (such as hypertension, coronary heart disease, diabetes, and stroke). The etiological basis of cardiovascular disease has been shown to begin with atherosclerosis. This study attempts to provide useful information for the prevention and treatment of early stage cardiovascular and cerebrovascular diseases by analyzing carotid atherosclerosis incidence and relevant traditional risk factors by gender and age groups. We developed a cutpoint-based strategy for preventing carotid atherosclerosis in different genders and age groups.

Atherosclerosis typically begins in childhood. Clinical manifestations become apparent with older age; however, in some cases, younger patients are affected. The first onset of coronary heart disease and stroke could be disabling or fatal. It is essential to manage the risk factors of cardiovascular and cerebrovascular diseases and to limit the exposure to risk factors, which could ultimately reduce the occurrence of cardiovascular and cerebrovascular diseases. Researchers [25], [26] have shown that a high TC level is an independent predictor of carotid atherosclerosis in elderly populations and that a high TG level greatly affects the development of carotid atherosclerosis in elderly women. Studies [27], [28] conducted by Sunjiayi showed that the occurrence of carotid atherosclerosis increases as the FBG level rises in women; however, it does not increase in men. These results might suggest that the role of FBG in carotid atherosclerosis is not identical in women and men. The results of our study are consistent with the study mentioned above, demonstrating that decision tree analysis could successfully screen for the important risk factors for carotid atherosclerosis in different genders and age groups.

This research is a cross-sectional study. Most of the subjects were from Hangzhou, where people have higher levels of FBG, TG and TC than those of the populations in poorer areas. Therefore, the results might not be generalizable to the populations in developing areas. There might be differences in the CIMT measurements and in the definitions of carotid atherosclerosis among institutions and countries. Finally, the relationship between traditional risk factors and carotid atherosclerosis has not shown statistical significance in individuals aged 60–80 years, regardless of gender, which might be a limitation resulting from the number of subjects or the involvement of non-traditional risk factors. Additional work should be conducted to investigate this issue.

## Conclusions

Carotid atherosclerosis is highly widespread in healthy populations. Traditional risk factors and relevant cutpoints are not identical in different genders and age groups. The specific gender and age group-based cutpoint strategy might contribute to preventing cardiovascular disease. Men should be concerned with TC (>6.31 mmol/L) from 20–40 years of age and FPG (>5.79 mmol/L) from 41–59 years of age. Women should be concerned with FPG (>5.52 mmol/L) from 20–40 years of age and TG (>1.51 mmol/L) from 41–59 years of age. We identified cutpoints for the risk factors, and future research should focus on the correct approach for reducing this risk.
